# Hospital and Institutional News

**Published:** 1915-11-06

**Authors:** 


### HOSPITAL AND INSTITUTIONAL NEWS.

### WAR EFFECTS ON VOLUNTARY HOSPITALS.

In our issue of October 23 we published a fairly complete survey of the general effect of the war upon a first list of typical metropolitan and provincial hospitals, including certain special and one Glasgow hospital. With a view to doing justice to all the London, provincial, Scottish, and Irish hospitals, we wrote to the representatives of twenty such institutions asking for necessary details and information. The answers are coming in, some of them most excellent, and we rely on the courtesy and grit of the secretaries and superintendents concerned to let us have full particulars with as little delay as possible. The approach of Christmas makes it desirable in the interests of these institutions that the further article shall appear before the end of the year, preferably, if full advantage is to be reaped, not later than the first week in December.

### A "NURSE CAVELL BED" AT CARDIFF HOSPITAL.

Sir William James Thomas, of Ynyshir, has forwarded a cheque for 1,000 guineas to Colonel Bruce Vaughan for the endowment of a bed in the King Edward VII.'s Hospital, Cardiff, in memory of Nurse Edith Cavell. In a covering letter Sir William makes it clear that he is hopeful other voluntary hospitals in the kingdom will raise similar memorials to this noble woman, and he is also desirous of recognising the loyal body of nurses who have contributed so largely to the success of the Cardiff hospital by associating permanently with the hospital the name of one who has shed a new and undying lustre upon a profession already so deservedly honoured. No more fitting memorial could have been thought of, as it will perpetuate in Cardiff for all time the mission of Nurse Cavell's life; and if the hospitals generally follow Sir William's example, many poor sufferers in days to come will have cause to be thankful for the life and death of this heroic Christian gentlewoman. It is particularly appropriate that wounded soldiers are at present accommodated in the ward selected for the "Cavell Bed," whilst it is gratifying to Sir William personally that the same ward already contains beds previously endowed by him to the memory of his grandfather and his father. Before taking any steps in the matter Sir William ascertained the wishes of Mrs. Cavell, who promptly wired him, "Very grateful for the privilege."

### BABIES ON EXHIBITION.

Queen Amelie of Portugal is to open an exhibition relating to "Women and Their Work" at Prince's Skating Club, on Monday, November 8, of which Queen Alexandra, Princess Arthur of Connaught, and the Duchess of Teck are patronesses. It is claimed that every woman who visits the exhibition will be provided with an opportunity for choosing some new career, and the occupations of which illustration is afforded include women's invalid kitchens, various village industries, a woman blacksmith at work, women police, a women's guard of honour, and women's service aircraft. All this is highly interesting and instructive. We regret, however, to note that among the exhibits it is proposed to provide the public with the spectacle of a "baby incubator, with babies, under the supervision of trained nurses." We do not know what institution is responsible for lending its patients on show, nor under whose authority trained nurses are withdrawn from duty at this unprecedentedly busy time to act as show-women. But we venture to protest against this abuse of specialised medical treatment for infants lacking in vitality. Persons seriously interested in studying this mode of rearing babies can obtain opportunities of doing so in maternity institutions. But ailing infants are entirely out of place in an exhibition catering for the amusement of the public no less than for its instruction.

### IMPROVEMENTS AT THE MIDDLESEX.

In war-time one looks for few developments at the old London hospitals except such as are rendered necessary by the war itself. The need for economy, the scarcity of labour, and, above all, the necessity for finding accommodation for wounded soldiers have combined to call a halt to structural improvements. At the London hospitals few new features are expected at present, unless they were well on their way before the outbreak of war. One such instance is the reconstructed children's ward at the Middlesex Hospital, formed by taking into the ward a long and spacious corridor; much more bed space has thus been afforded, while the facilities for ventilating and lighting the ward have been improved. The sanitary tower in connection with the wards of the south-west wing has also been opened. The erection of this tower was delayed in the first instance by trade disputes, and later by the war, but by its completion the service of the wards in question has been greatly improved, since provision is made for a new bathroom and lavatory for each of the wards. The rebuilding of the north wall of this hospital has been recently completed. The work of rebuilding had to be carried on in such a way as to interfere as little as possible with the work of the institution, a fact which increased the difficulties experienced by the builders and the hospital's officers. A further improvement at the Middlesex is to be found in the surgical wards, the walls of which are now tiled. The combined effect of all these alterations is good, and the hospital authorities are to be congratulated on securing their completion.

### THE PRACTICES OF DOCTORS WHO ARE AT THE FRONT.

Sir Donald MacAlister's presidential address at the opening session of the General Medical Council on November 2 contains some plain speaking on the subject of "free choice of doctor" under the Insurance Act, and on the kindred topic of arrangements between practitioners with respect to private patients. Sir Donald even suggested that failure of a practitioner to observe any arrangement made with an absent colleague concerning the latter's patients would be "regarded as dishonourable by his professional brethren of good repute and competency," and would therefore form a proper subject of inquiry by the Council. We quite agree with this pronouncement, but we are also certain that the subject is very thorny, and not to be tackled without great caution. For instance, there are many Territorial medical officers who have already been away from their practices for fifteen months. Supposing a doctor who enlists now has his practice protected by some arrangement such as that foreshadowed by the President of the Medical Council; it may easily happen that since the war began he may have obtained many new patients from among the *clientèle* of his Territorial neighbour. Is he to get that protection in respect of such patients which his colleague who has borne the burden and heat of the day has failed to receive? One could suggest a dozen such knotty points for consideration, and it is to be hoped the Council will walk warily in this matter.

### AN EXTRAORDINARY CASE AT BRADFORD.

Some not very complimentary criticisms of the management of the Bradford City Hospital were heard at an inquest in that town on October 26, and we must say that even stronger strictures would not have been out of place. The facts elicited were, briefly, that a child of a few days old, under treatment as an in-patient for some eye disease, was burnt by a hot-water bottle in mysterious circumstances, and died thereof about three weeks later. The Coroner's efforts to fix the responsibility for this regrettable occurrence were not very successful, and straightforward evidence of the incident seemed impossible to come at. This, however, was not the most extraordinary feature of the case. For the fact of the child having been burnt was concealed from the parents until three days before its death: presumably, that is, until the child was seen to be moribund. This concealment was practised at the instance of the medical officer in charge, whose conduct was injudicious in the extreme. It was shown that three large burns existed, and from the dimensions given it is clear that the prognosis was grave from the start, which makes the medical officer's conduct all the more foolish. Yet another unsatisfactory feature of the case was that although the burns were detected at 3.30 in the afternoon, they were not reported to the medical officer until the next day. The managers of this hospital have a house that badly needs setting in order.

### A GOOD EXAMPLE.

An old custom has been abandoned in Lancashire, and one we should like to see treated with the same disrespect in every county in the kingdom where it still exists. For the remainder of the year the High Sheriff of the shire (Mr. Edward Graham Wood) has decided to discontinue the official dinners at which it is customary for the High Sheriff to entertain the Grand Jury, and instead to make a contribution to the funds of the Red Cross Society and the Order of St. John of Jerusalem. In sending a cheque for £500, Mr. Wood has stated that he regards economy in both public and private life as a first obligation. We earnestly trust that his fine example will be largely followed, and we greatly admire the spirit which has dictated his generous conduct.

### THE WAR AND THE EASTBOURNE GUARDIANS.

Some little time back the Army Council asked the Eastbourne Guardians to make the local workhouse available as a military hospital. The Guardians agreed at once and removed the inmates. At the last meeting of the Guardians a letter from the Army Council was read regretting that it would not be found possible to utilise the workhouse owing to the heavy cost of adaptation. The reading of the letter aroused, not unnaturally, a great deal of indignation. It is certainly strange that the Army Council did not assure themselves of the suitability of the buildings before asking that they might be placed at their disposal. Although the letter seems perfectly clear, the Clerk to the Guardians suggested that there must be some mistake, and that the Council, though it did not want the workhouse as a whole, did want the infirmary part of the buildings. The Guardians decided to clear this point up before returning the former inmates. The question of the responsibility for the bungling which has occurred is to be raised in Parliament by Mr. Gwynne, M.P. for the town.

### PROFESSOR CARREL'S RESEARCHES.

For a year or so Professor Carrel, of the Rockefeller Institute in New York, has been in charge of an experimental hospital in France provided by the generosity of Mr. Rockefeller. This hospital is behind the French lines, somewhere in the Compiegne district, and is staffed by several noted experts in particular branches of scientific research. For many months the work has been devoted to finding some antidote for those distressing cases of "gas-gangrene," caused by the *bacillus aerogenes capsulatus* and other deadly organisms, which are so fertile a source of death or complete disablement. It would seem as if this research has proceeded along lines somewhat divergent from those which Sir Almroth Wright has been following in this country, and more nearly approaching those of Sir Watson Cheyne: that is, it has been along antiseptic rather than auto-sero-therapeutical channels. A great number of antiseptic substances have been experimented with, and rejected for one reason or another; but some few have come out of the scrutiny with honour, and the first place is allotted to a mixture of sodium carbonate with bleaching powder (in solution), acidified by the addition of boric acid. This liquid is said to have great antiseptic action, while not detrimental to the tissues; further, it has strongly solvent properties on necrotic tissues, and penetrates well into the depths of the wounds.

### SELF-INOCULATION OF "GAS GANGRENE"

Some two or three weeks ago a sensational paragraph in the more excitable sections of the daily Press announced that a (female) assistant at the American Ambulance in Paris had voluntarily inoculated herself with a virulent culture of *B. aerogenes capsulatus* (the commonest micro-organism of the deadly "gas-gangrene"), in order that the efficiency of a certain remedy (quinine hydrochloride) might be tested. The lady had done this without the knowledge of the research staff, merely reporting the deed two hours later. She had injected one dose deeply into the muscles of the left thigh, and another just under the skin of the right thigh. Quinine was at once injected into the left thigh through a needle pushed down as far as possible to the same place as where the bacilli had been deposited, according to the patient's indications. No treatment was adopted for the right thigh. Except for a temporary local swelling of the left thigh and a slight constitutional disturbance she recovered very rapidly. In reporting the facts to the *Lancet*, Dr. Taylor, the pathologist to the ambulance, will not commit himself to an opinion whether the remedy had anything to do with the cure, or whether the resistance of the individual was responsible.

### THE M.A.B. AND MATRIMONY.

The Metropolitan Asylums Board have just discovered that nearly 300 of their nurses have got married, mostly to soldiers, without official permission. The nurses have all been formally dismissed, but have been re-engaged subject to the condition that their services can be dispensed with at one day's notice. It is, no doubt, reprehensible that these nurses should have disobeyed the regulations as they did; and in ordinary times the infraction of discipline might have involved more serious measures. But in the universal upheaval caused by war it is so desirable that matrimony should be encouraged that the Asylums Board are well advised to be lenient; and we should even like to see a temporary suspension of regulations of this kind, under such safeguards as will protect the Asylums Board and similar governing bodies from detriment to the interests of their patients.

### A HEAVY DEFICIT AT SHEFFIELD.

At the annual meeting on October 20 of the governors of the Sheffield Royal Hospital a very satisfactory report of the year's working in every respect but that of finance was submitted. A large number of both in-patients and out-patients received treatment; the former reached a total of almost 3,500, nearly 300 more than in the previous year, and about the same as the high figure of two years ago. A large number of wounded soldiers have been under treatment, and the Lord Mayor spoke somewhat wistfully of the possibility of the charity being reimbursed out of public funds for the expenses thus incurred. Financially, the year was marked by a sad deficiency in legacies, which brought in but £811. The annual income went up £312 to £11,519; and expenditure £589 to £17,769. By taking in legacies the deficit on the year was reduced to £5,435, but even this figure is sufficiently alarming. In a business community such as Sheffield these figures are certain to attract attention, and we sincerely hope there will be a better showing twelve months hence, when the next annual report comes to be presented. To secure this the real cause of this falling off in funds should be ascertained and remedied.

### THE URGENCY CASES HOSPITAL.

In an eloquent appeal for funds on behalf of the hospital which is working behind the French lines in the Argonne, and bears the above title, Mr. Stephen Paget has made public some interesting particulars about the work that has been accomplished. The hospital takes only those who have been severely wounded—in the French phrase, *les blessés qui peuvent mourir*—and hence the quantities of dressings required are very large. The French authorities have asked for an increase from 120 to 200 beds, which shows that the Urgency Cases Hospital is appreciated by our Allies. In spite of the serious nature of the admissions and the proximity (ten miles) of the hospital to the firing-lines, the death-rate has been no more than 4 per cent. For want of funds to build with, a chapel has had to be fitted up as the operating-theatre, a piece of apparent vandalism which the committee would much like to abandon if their financial resources permitted them to do so. The working expenses of the hospital are about £100 per week.

### THE BUILDING SCHEMES OF LOCAL AUTHORITIES.

The vast importance of economy of all kinds by local authorities in the present crisis, and especially of avoiding new building schemes, does not seem to have come home to some of these bodies. Only a few days ago a proposal was put before the Staffordshire, Wolverhampton, and Dudley Joint Tuberculosis Committee for proceeding at once with the plans of the projected Lower Penn Sanatorium at a cost of £15,000, or £150 per bed. We are glad to say that the proposal was rejected. A peculiar feature of the incident, as reported in the *Wolverhampton Express and Star*, was that the Clerk was the introducer of the proposal and spoke in favour of it, whereas the members of the committee who discussed the matter seemed never to have authorised or even considered the high figure of expenditure upon which the Clerk wished to embark them. We agree that £150 per bed is too high, and also that, except to meet admitted and very urgent necessities, all such schemes as this should be indefinitely postponed.

### CHARGE FOR VISITORS' TEAS.

At one of our large hospitals for incurables visitors are admitted daily from 2 to 6 p.m., and on Sundays from 2 to 4 p.m. Since the institution was founded, over sixty years ago, it has been the custom for patients to be allowed to invite visitors to take tea with them. Many of the visitors—if not the majority—belong to the well-to-do classes. Of recent years the number of visitors has increased, and daily quite a large number take tea. In view of the fact that the cost of provisions has increased of late, the house committee have decided to charge each visitor who takes tea on the hospital premises the sum of threepence. In future no tea will be served to a visitor unless a ticket is presented, and tickets are only obtainable at the entrance lodge. It will be informing to note whether the number of visitors taking tea decreases in consequence of the new rule, and it will be interesting to see what sum of money at the end of twelve months is received as a result of the charge of threepence per head.

### THE PRICE OF COMMODITIES IN WAR-TIME.

In the fiftieth annual report of the Glamorgan County Lunatic Asylum are contained some interesting comparisons between the prices, contract and other, paid before and after the outbreak of war. Thus, to take the average prices paid in 1913 and in the first quarter of 1915, we find that some articles were actually cheaper than before the war: thus, English beef and mutton, bacon, and mustard were actually obtained at lower prices than those paid in 1913; while for soda, starch, pepper, snuff, and tobacco the prices ruling showed no variation. For most commodities, however, there are more or less substantial increases to record. Frozen meat is up about 50 per cent.; butter, 20 per cent.; margarine, 14 per cent.; flour, 50 per cent.; sugar, over 60 per cent.; oatmeal, about 35 per cent.; currants, 7 per cent.; and rice, only 1.4 per cent. Split peas have jumped to just twice the old price. Soft soap has done the same, but yellow soap is only about 7 per cent. dearer than formerly. We doubt whether any institution in Germany could show such small increases!

### THE DONCASTER INFIRMARY REPORT.

The annual statement to the governors of this institution discloses a very satisfactory condition of activity, though the financial situation might be better. During the year 887 in-patients were treated in 65 beds, a large increase on the previous year. Thanks to a slight increase of income, and a large decrease of expenditure, the deficit on the year's work was £274, as against £666 the year before. By absorbing legacies to the value of £770 the total debt at the bank was reduced to £644; but this method of treating legacies is not exactly ideal, as Archdeacon Sandford had the courage to acknowledge in his speech. The building fund for a new infirmary has reached just over £1,000, and the Mayor announced that after the war a determined effort would be made to rebuild. Certainly the committee are wise not to entertain the notion at present; and, for war-time, they are to be congratulated on the management of their institution and the wide basis of support which they attract.

### THE UNPAID CHARITABLE WORKER.

Those who read the paragraph which we published in our issue of October 23 on "The Economics of Unpaid Employment" will realise the difficulties which surround the manufacture of hospital garments, bandages, dressings, etc., by unpaid voluntary workers, who by their well-intentioned activities may be taking away the livelihood of those who have no other means of living than the making of this class of materials. To show how much voluntary work is expected to be done at Burlington House, one wing of which, as we recorded last week, has been handed over to the British Red Cross Society for use as central workrooms, it is only necessary to record that those who wished to register there before its opening were allowed to do so if they were willing to devote five mornings or afternoons a week to the work. Now that winter will soon be upon us, we must expect those who represent the interests of the workers in this industry to be raising their voices on their behalf, since no poor worker can hope to live in competition with the unpaid charitable worker.

### A HOSPITAL WITH A WEAK SPOT.

The annual meeting of the Birmingham Orthopædic and Spinal Hospital was held last week, and the chief points brought out were the increased calls likely to be made on the institution in treating injured soldiers, and the need for pushing forward with the rebuilding scheme. Reference was made last week in our columns to the difficulties of special hospitals, and doubt was expressed as to the possibility of this institution being able to secure the necessary funds for a rebuilding scheme. At the above meeting the President, Mr. Ansell, alluding to the financial position, said that the deficit for the past year was £800, a sum less by £320 than that for the previous year. It appears that the weak spot is the subscription list, which is altogether inadequate to the needs of the institution, and disproportionate to its annual expenditure. The Birmingham Orthopædic Hospital, by virtue of its claim to be the only special hospital of this class outside the Metropolis, should be in a position to make a strong appeal to residents in the Midlands. If the rebuilding scheme has perforce to be held up for the present, there can be no reason for delaying those efforts, which seem so urgently called for, which will remedy the very unsatisfactory state of affairs in connection with the subscription list. Weakness in this direction is weakness indeed in provincial hospital finance.

### THE STAGE AND THE RED CROSS.

The proverbial generosity of theatrical managers and performers where charitable funds are concerned has been many times exemplified already during this war. The latest instance of this energy in well-doing comes from the "movies" and those who have "played for the film." On November 9 a large number of picture playhouses are handing over their day's takings to a special fund for the provision of a complete motor ambulance convoy, for which purpose it is hoped to raise £30,000. The public have been requested to patronise on that day only those establishments which are thus helping the Red Cross—a recommendation laudable enough, but not likely to be followed, we fear. Then, on November 16, there is to be a special matinée at the London Opera House, at which only those artists will perform who have already acted for the cinema. The list includes many of the best-known actors and actresses now before the public, and the matinée should prove highly successful.

### HOSPITAL SUNDAY IN BIRMINGHAM.

The appeal of the Lord Mayor to the citizens of Birmingham on behalf of the Hospital Sunday movement is one which should prove stimulating to the inhabitants of that great city. Last year's Hospital Sunday collection was below the average, and it would be trebly gratifying to learn that on Sunday last the response was such as to create a new and higher average and to relieve substantially the increasing financial burdens which threaten seriously to restrict the beneficent activities of the Birmingham hospitals. Coincident with diminished incomes, the hospitals are faced with heavier expenditure, due to the higher cost of drugs, dressings, and provisions. Their difficulties and the urgency of their needs must increase as time passes, and after the war the demands on their accommodation are likely to increase rather than to diminish.

### THE AUTUMNAL FEVER EPIDEMIC.

It is quite in accordance with the usual order of things for the fever statistics to show increases just now. In point of fact, there is a distinctly more pronounced increase this year than usual in London, and it is to be hoped that the epidemic will make no further headway. Over 450 cases of scarlet fever and diphtheria were admitted in four days into the Metropolitan Asylums Board hospitals recently; and the total number of patients under treatment rose to over 5,000. No one knows, or has any plausible hypothesis, why this autumnal prevalence of these fevers should occur with such regularity; when the biology of scarlet fever is placed upon a more satisfactory foundation possibly research may be able to throw some light upon its epidemiology.

### MANCHESTER ROYAL INFIRMARY.

At a meeting of the Manchester Royal Infirmary Board on October 27, Dr. William B. Anderton, who has been pathological registrar at the Royal Infirmary for twelve years, and lecturer on pathology at Manchester University, was appointed to the post of honorary pathologist at the infirmary. Mr. G. B. Warburton, F.R.C.S. (Eng.), was appointed resident surgical officer.

### HIGHER COST OF PATIENTS' MAINTENANCE.

At the last meeting of the Aberdeenshire Sanatorium Benefit Committee, a communication was received from the managers of the Thomas Walker Hospital, Fraserburgh, asking for payment by the Committee of an increased charge of 2s. 6d. per week in respect of each patient sent. The letter stated that the patients, being phthisical, were exceptionally costly, part of their treatment consisting in the provision of abundance of nourishing food. The Sanatorium Benefit Committee is not disposed to comply with the request without protest. It appears that in addition to the patients in the hospital proper, there are five shelters in the grounds which have been erected at the expense of the Committee, and the managers are being paid 30s. per week in respect of the patients in these shelters, which is the same as for the patients in the hospital. The Committee has decided to ask the managers to take these facts into consideration. It is undeniable that the cost of maintenance of patients—especially of tuberculosis patients—is increasing at a serious rate, and it is difficult to see how Sanatorium Benefit Committees can avoid additional expense. Tuberculosis work all over the country is bound to be hampered by the increased cost of food.

### INFECTIOUS DISEASES AND VERMIN.

Some interesting facts are given by Dr. G. B. Millson, medical officer of health for Southwark, in his annual report, relating to the propagation of measles, scarlet fever, and kindred diseases by such vermin as bugs, fleas, and lice. For years he has thought such vermin have played a very important part in the spread of these diseases, and much of the sanitary work of the borough is directed to ridding the houses of the pests. The houses were cleaner than they were years ago, yet there was still in that respect much to be done. The verminous state of the houses was, in his opinion, the cause of the spread of scarlet fever, from which the inhabitants suffered during the last year, and municipal cleansing of the home and person in the poorer districts appeared to be the most economical treatment in the prevention of these diseases. The figures in the report show how thoroughly the sanitary staff, as well as the teaching staffs in the numerous London County Council schools, have done their utmost to get a thoroughly cleanly people. During the year 2,755 persons were cleansed at the Council's station, as against 2,604 in the previous year. Of these 2,662 were children, and in a large number of cases it was found necessary to treat the children twice, and sometimes three or four times, before they were found to be effectually cleansed. From the common lodging-houses, and other places in the borough, ninety-three adults were sent to the station. In addition, the sanitary staff under Dr. Millson's charge had to deal with the cleansing of dirty rooms and their contents, 1,371 being treated by the occupiers and owners, and 1,292 by the staff. Much good work is done in this direction by the officials, work which is little understood by those living in clean, decent homes in less congested districts.

### THE STRAIN ON SMALL HOSPITALS.

The recently retired chairman of the Gordon Hospital for Rectal Diseases, in the Vauxhall Bridge Road, has addressed a letter of appeal to the newspapers urging the claims of this institution for assistance in relieving the strain which is severely felt at present by small hospitals. Mr.

H. F. Wilson, who has been succeeded in the chairmanship by Mr. Beasley Robinson, was able, during his year of office, to reduce the burden of the mortgage debt from £4,500 to £3,700, but the outbreak of war precluded any hope of carrying the process further. An appeal is now made for funds to enable this hospital to continue its work of alleviating the very distressing complaints with which it deals. The Gordon Hospital was founded in 1884, and was then called the Western Hospital for Fistula; its present name was attached to it in 1886.

### EXCHANGING CHARITY VOTES.

At many orphanages and similar institutions where the voting system is still in practice a great deal of work and trouble is experienced by those who have charge of the correspondence. It appears that when any kindly person is interested in the candidature of an applicant offers of votes for other institutions than that in view are received. The only way in which to deal with these votes is to endeavour to exchange them for those of the charity to which admission is sought by the particular case. A worker will write to the secretary of an institution and say that she has received a number of votes for such-and-such a place, and she is anxious to know whether they can be exchanged for her. So great has the feature become of late that one well-known institution has printed the following message in the form of a circular, and is sending it to all those who write letters on the subject: "The secretary presents his compliments and begs to state that the board of management of this hospital do not recognise any *exchange of votes*. It is not helpful to a candidate who is accepted for the benefits of this hospital to send here votes issued by another charity, and the secretary cannot undertake to effect any exchange on behalf of interested persons." We are glad of this attempt to destroy a practice which has been greatly abused in the past.

### THE WELSH MEDICAL SCHOOL.

At the annual meeting of the Court of Governors of the University College of South Wales and Monmouthshire, held recently at Cardiff, Principal Griffiths paid a striking tribute to the donor of the proposed Medical School buildings, Sir William James Thomas of Ynyshir. (See The Hospital, October 23, 1915, p. 66.) The Principal said that he did not think they sufficiently appreciated the gift of Sir William, which was the greatest that had ever been conferred on Wales by one man. In a letter he (Sir William) had expressed his great disappointment, because he thought that the matter would all have been settled before the war started. All he wanted was to serve his country, and he was prepared to make any sacrifice, but he was somewhat confused as to his duty, because he understood it was of immediate national importance that adequate facilities should be provided for training medical students. A resolution submitted by the Council was unanimously adopted, to the effect that in view of the pressing necessity of proceeding forthwith with the matter of the National Medical School the Council hoped it might be found possible to expedite the proceedings of the Commission, with the view of obtaining an early decision with regard to the Medical School and all other matters in relation thereto. They also expressed the hope that the Treasury would grant permission to the donor to proceed forthwith and complete the building of the Medical School.

### THE ROYAL SOUTH HANTS AND SOUTHAMPTON HOSPITAL.

At a meeting of the governors of this hospital, held on October 19, Colonel E. K. Perkins, D.L., J.P., was unanimously elected chairman of the hospital in the place of Mr. Courtenay E. Wilson, J.P., deceased. A vote of sympathy and condolence with the relatives of the late chairman was passed in respectful silence, after Colonel Perkins and Colonel Willan had rendered tribute to the able services of Mr. Wilson and the great advantage which the institution had derived from them. Colonel Perkins has been vice-chairman of the hospital for a considerable time, and is a Southampton man. He knows, according to Mr. Gayton in proposing his election, the work and the requirements of the hospital thoroughly. He is also an energetic member of the local Territorial Force Association, and has the support of all sections of the governors in his new office.

### THIS WEEK'S DRUG MARKET.

Again quinine has been the feature of the week, and the price has advanced to quite fantastic figures. It is really very difficult to understand why operators should be willing to pay such extraordinary prices when there appears to be no suggestion of shortage of the drug or of the raw material—cinchona bark. It is true that of late the consumption has greatly increased, and that the available sources of supply have become more restricted, but, nevertheless, there does not appear to be any reason to expect a famine in quinine, although from the excited state of the market one would imagine that a famine was imminent. Menthol has advanced substantially in price, and a good business has been done; it is rumoured that one of the reasons for the increased demand is that it is intended to supply our troops in the trenches with a warming beverage containing menthol as one of its ingredients, but we have not been able to confirm this. Oil of peppermint is also dearer. The values of the synthetic drugs are, on the whole, well maintained, and the prices of phenacetin, barbitone, and the sodium salicylates have an upward tendency. Supplies of acetyl-salicylic acid are somewhat more plentiful, and it is not impossible prices may tend downwards, although any substantial reduction is not to be expected, for the present at any rate. The high value of opium is well maintained. Eucalyptus oil is in good demand, and the value has an upward tendency. Further business has been done in cod-liver oil, and there are no signs that prices are likely to be lower.

